# TRPV4 regulates migration and tube formation of human retinal capillary endothelial cells

**DOI:** 10.1186/s12886-018-0697-2

**Published:** 2018-02-12

**Authors:** Lei Wen, Yue-Chun Wen, Gen-Jie Ke, Si-Qin Sun, Kai Dong, Lin Wang, Rong-Feng Liao

**Affiliations:** 1 Department of Ophthalmology, The First Affiliated Hospital of Anhui Medical University, 218 Jixi Road, Hefei, Anhui 230022 China; 2 Department of Ophthalmology, Anhui Provincial Hospital, Hefei, Anhui China

**Keywords:** TRPV4, HRCEC, Migration, Tube formation

## Abstract

**Background:**

Ca^2+^ entry plays an important role in modulating endothelial cell migration and tube formation. Transient receptor potential cation channel subfamily V member 4 (TRPV4) is a Ca^2+^-permeable channel that is widely expressed in endothelial cells. It has been reported that TRPV4 is expressed in HRCECs and regulates Ca^2+^ entry. However, the function of TRPV4 in human retinal capillary endothelial cells (HRCECs) remains unknown.

**Methods:**

In this study we used western blot and immunostaining assay to verify TRPV4 expression in HRCECs. And then we pretreated HRCECs with HC067047 and transfected with specific shRNA of TRPV4. The functional presence of TrpV4 was determined by using fluorescence, migration and tube formation assay in TrpV4 knockdown cells or control cells.

**Results:**

Using western blot and immunostaining, we confirmed TRPV4 expression in HRCECs. Moreover, inhibition of TRPV4 using the specific inhibitor HC067047 and the knockdown of TRPV4 with shRNA significantly suppressed tube formation and migration by HRCECs.

**Conclusions:**

TRPV4 is essential for HRCEC migration and tube formation, and maybe a potential therapeutic target for retinal vascular diseases.

**Electronic supplementary material:**

The online version of this article (10.1186/s12886-018-0697-2) contains supplementary material, which is available to authorized users.

## Background

As a second messenger, Ca^2+^ plays important roles in cellular functions such as migration, tube formation, and proliferation [1]. To date, > 30 transient receptor potential (TRP) channels have been studied. They are divided into seven subfamilies: TRPA (ankyrin), TRPC (canonical), TRPV (vanilloid), TRPM (melastatin), TRPML (mucolipin), TRPP (polycystin), and TRPN (NOMPC-like) [1, 2]. They are Ca^2+^-permeable, nonselective cation channels, which are widely expressed in endothelial cells (ECs) [3, 4]. They can be activated by a wide variety of stimuli (osmotic, mechanical, and chemical). Now, increasing research has shown that TRPV4 is important in regulating Ca^2+^ influx and plays vital roles in endothelial function. For example, flow-induced TRPV4 activation in lung ECs leads to lung permeability edema [5]; TRPV4 activated by H_2_S in mouse aortic ECs regulates vasodilation [6]; TRPV4 plays a minor role in controlling endothelial progenitor cell proliferation [7]; and H_2_O_2_ induces Ca^2+^ entry via TRPV4 in lung microvascular ECs [8].

Recently, several studies have focused on the function of TRPV4 in the retinal microvascular endothelium of rats and mice [9]. However, human-derived ECs have not been studied. During retinal development, the activation and migration of ECs are important in forming tubular structures. However, retinal neovascularization is also a characteristic pathological consequence of many retinal diseases, including retinopathy of prematurity [10], diabetic retinopathy [11], and retinal vein occlusion [12].

In previous studies, we showed that TRPV4 regulates flow-induced endothelial Ca^2+^ entry [13] and vascular function [14]. Besides, the TRPV4-KCa2.3 signaling pathway plays an important role in smooth muscle hyperpolarization and relaxation and may be involved in endothelium-derived hyperpolarizing factor dysfunction in diabetic rats [15]. Here, we show that TRPV4 is expressed in human retinal capillary ECs (HRCECs) and is essential for their migration and tube formation.

## Methods

### Cell culture

Human retinal capillary endothelial cells (HRCECs) (BeNaCultureCollection, Wuhan, China) and human embryonic kidney (HEK) 293 cells (ATCC, Manassas, VA) were used between passage 10 and 15 and maintained in Dulbecco’s modified Eagle’s medium (DMEM) (Gibco, Gaithersburg, USA) supplemented with 10% fetal bovine serum (FBS)(Sijiqing, Hangzhou, Chna) with 100 U/mL penicillin and 0.1 mg/mL streptomycin (Beyotime, shanghai, China) at 37°C in a humidified incubator under 5% CO_2_ [16].

### Western blotting

HRCECs and HEK293 were harvested and washed three times in phosphate-buffered saline (PBS). The cells were lysed in RIPA buffer (P0013C, Beyotime, shanghai, China) supplemented with 1% phenylmethylsulfonyl fluoride (Beyotime, shanghai, China) to obtain the proteins, which were separated on SDS-PAGE (10%), and transferred to PVDF membranes. The membranes were incubated with primary antibodies against TRPV4 (1:200, Alomone, Israel) [17] and β-actin (1:3000)(santa cruz biotechnology, California, USA) at 4°C overnight. Then the membranes were incubated with HRP-conjugated secondary antibodies for 2 h at room temperature. Finally, the blots were developed with an enhanced chemiluminescence reagent (P0018, Beyotime). Images were analyzed using ImageJ (National Institutes of Health, Bethesda, MD) [16].

### Immunostaining assay

HRCECs were fixed in 4% paraformaldehyde for 30 min and permeabilized with 0.1% Triton X-100(Invitrogen, Grand Island, NY, USA) for 10 min at room temperature. After blocking with 5% bovine serum albumin in PBS for at least 30 min at room temperature [18], the cells were incubated with specific primary antibodies against TRPV4 overnight at 4°C, and then with Alexa Fluor secondary antibodies(Invitrogen, Grand Island, NY, USA) for 2 h at room temperature. Cells were washed five times in PBS after each step. The images were captured using a confocal microscope.

### Ca^2+^ level measurement

HRCECs,which were grown to 80% confluence, were first pretreated with or without 10 μM HC067047 for 30 min. After that, 10 μmol/L Fluo-4 was loaded into them. Then, the cells were stimulated with 1 nM, 3 nM, 10 nM, 30 nM, 100 nM GSK101670A (G0798, Sigma, Deisenhofen, Germany) and the fluorescent signals were recorded every 7 s using a fluorescence imaging system (IX71 inverted microscope, Olympus) [19].

### Migration assay

Transwell chambers with a 0.4-μm pore size membrane. Cells were seeded at 3.8 × 10^4^ in 100 μl serum-free DMEM in the top of a transwell chamber (353,097, corning, NY, USA) [20]. DMEM with 10% FBS (100 μl) was added to the lower chamber. After 48 h incubation at 37°C in a humidified atmosphere of 5% CO_2_, cells migrated to the lower surface of the chambers. And then, the chambers were removed and washed twice in PBS. The cells were fixed in 4% paraformaldehyde for 30 min and stained with crystal violet. Non-migrating cells were removed from the upper chamber with cotton-tipped swabs. Images were captured with a video camera (Coolpix 54, Nikon, Japan).

### Tube-formation assay

50 μl Matrigel (354,234, BD Bioscience) was added to each well of a 96-well plate and incubated for 30 min at 37°C in a humidified atmosphere of 5% CO_2_ [19]. Until matrigel was polymerized, Cells (4 × 10^4^ in 100 μl DMEM) were seeded in each well. Capillary-like tubes were formed within 8 h and recorded with the video camera (Coolpix 54, Nikon, Japan).

### Cell transfection assay

HRCECs for infection were plated in 6-well plates, after 24 h, 100 μl lentivirus-3(LV3) packed LV3-TRPV4 shRNAs (GenePharma, Suzhou, China) were added to the cells. After 3 days, the cells can be used for further experimental study. HEK293 were transfected with empty vector and TRPV4 plasmid, using Lipofectamine 2000 (Invitrogen, Grand Island, NY, USA) as previously described [13, 21].

## Results

### TRPV4 expression in HRCECs

**Fig. 1:**
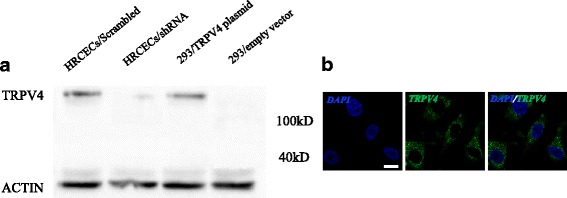
TRPV4 is expressed in HRCECs. **a** Western blot analysis. **b** Immunofluorescence using confocal microscope. Shown are representative nuclear DAPI staining (Left, blue), TRPV4 staining (center, green)merged images (Right). scale bar: 25 μm

To confirm that TRPV4 is expressed in HRCECs, we performed western blots (Fig. 1a) and immunostaining assays (Fig. 1b) [22] on cell lysates of HEK293 cells and HRCECs. All the data showed that HRCECs express TRPV4.

### TRPV4 regulates Ca^2+^ entry into HRCECs

**Fig. 2:**
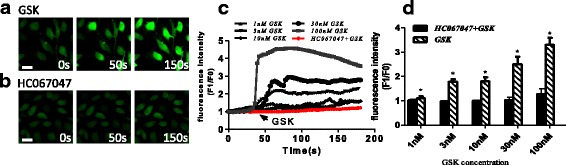
TRPV is important for GSK-induced Ca^2+^ influx. **a**, **b** Representative Ca^2+^ images of HUCECs stimulated by GSK with (**a**) and without (**b**) HC067047 treatment. **c** Representative images of of time course of intracellular Ca^2+^ levels (F1/F0). **d** Quantification of time course of intracellular Ca^2+^ levels (F1/F0) as in A and B (*n* ≥ 6). (scale bar, 25 μm). Mean ± SEM, (n ≥ 6; *p < 0.05, compared with HC067047 + GSK)

Our previous experiments had demonstrated that TRPV4 is expressed in HRCECs, so we then investigated its function using Ca^2+^ measurements. We found a robust increase in intracellular Ca^2+^ levels after stimulating cells with 100 nM GSK (a specific TRPV4 agonist) (Fig. 2a, c), while pretreatment with HC067047 (a specific TRPV4 antagonist) [23] significantly inhibited this effect (Fig. 2b, c). These data showed that TRPV4 is important in GSK-induced Ca^2+^ influx into HRCECs.

### Effect of TRPV4 on HRCEC migration

**Fig. 3:**
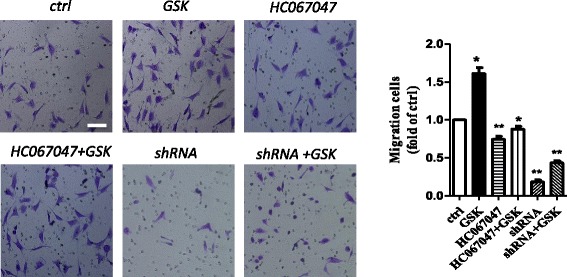
TRPV4 plays a role in HRCEC migration. Left panels: representative images of migration assays of HRCECs (scale bar, 25 μm). Right panel: data analysis. Mean ± SEM, (*n* = 6; *p < 0.05, ^**^p < 0.01 compared with control)

To evaluate the role of TRPV4 in HRCEC migration, we performed transwell assays with or without a TRPV4 inhibitor. The results showed increased Ca^2+^ influx in GSK-stimulated HRCECs (Fig. 3). However, this effect was blocked when HRCECs were pretreated with the specific TRPV4 inhibitor HC067047 and the specific shRNA. These results demonstrated that TRPV4 plays a key role in the migration of HRCECs.

### Effect of TRPV4 on HRCEC tube formation

**Fig. 4:**
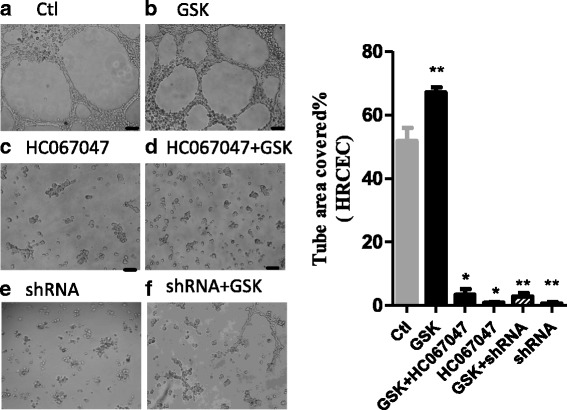
TRPV4 is necessary for tube formation. HRCECs were seeded on matrigel and visualized 8-12 h late. **a** In control condition. **b** Treated with GSK. **c** treated with HC067047. **d** treated with HC067047 and GSK. **e** treated with shRNA. **f** treated with shRNA and GSK. Data analysis shown in the right. (scale bar, 200 μm). Mean ± SEM, (*n* ≥ 10; *p < 0.05, ^**^p < 0.01 compared with control)

In addition to migration, we studied the role of TRPV4 in tube formation by HRCECs. As expected, GSK increased tube formation (Fig. 4b), while pretreatment with HC067047 and shRNA blocked the effect (Fig. 4c, d). The results showed that almost no tubular structures formed after co-incubation of HRCECs with HC067047 and GSK, and also we verified the same results with HRCECs, which were transfected with specific shRNA of TRPV4. (Fig. 4f, g). All these results suggested that TRPV4 is involved in tube formation by HRCECs.

## Discussion

In this study, we assessed the role of TRPV4 in regulating HRCEC migration and tube formation. Using western blotting and immunostaining, we first confirmed that TRPV4 is expressed in HRCEC. The results of Ca^2+^ imaging demonstrated that TRPV4 regulated Ca^2+^ influx in HRCECs. By using the specific TRPV4 inhibitor HC067047 and using shRNA, we showed that inhibition or knockdown of TRPV4 significantly suppressed migration and tube formation by HRCECs.

Recently, several studies have demonstrated that TRP channels play vital roles in retinal epithelial, corneal, and endothelial cells. For instance, the decreased level of TRPV4 induced by hyperglycemia and diabetes contributes to diabetes-induced endothelial dysfunction and retinopathy in retinal microvascular endothelium [9]. Thermo-sensitive TRPV4 activation protects human corneal ECs [24]. Activation of TRPV4 modulates the extracellular melatonin in human non-pigmented ciliary ECs [25]. Activation of TRPV4 is necessary for the correct establishment of tight junctions in corneal epithelia as well as the regulation of both the barrier function of tight junctions and their ability to respond to epidermal growth factor [26]. However, no studies had focused on TRPV4 function in HRCECs. This is the first study, to our knowledge, to examine the role of TRPV4 in regulating HRCEC function.

It is well known that TRPV4-regulated Ca^2+^ influx is important for endothelial migration and tube formation, which are vital components of angiogenesis [27]. Also, dysfunctional angiogenesis may lead to several retinal diseases [1, 28]. For example, functional expression of TRPV4 in retinal microvascular ECs are reduced in hyperglycemia and diabetes [9]; activation of TRPV4 in retinal ganglion cells leads to glaucoma and retinal detachment [29]; and TRPV4 is a target for treating ocular hypertension and conventional outflow by regulating Ca^2+^ homeostasis and cytoskeletal remodeling [30]. These studies inspired us to test the hypothesis that TRPV4 is associated with human retinal diseases and can it be a potential therapeutic target.

## Conclusions

In summary, we have provided direct evidence of functional TRPV4 expression in HRCECs and demonstrated that TRPV4 is involved in migration and tube formation in HRCECs.

## Additional file

Additional file 1: Raw materials of western blot, what I used is framed in the red box. Raw materials of transwell and tube formation, every first picture is what I used in the article. (PPTX 10465 kb)

## Abbreviations

DMEM: Dulbecco’s modified Eagle’s medium
ECs: Endothelial cells
FBS: Fetal bovine serum
HEK293: Human embryonic kidney 293 cells
HRCEC: Human retinal capillary endothelial cells
PBS: Phosphate-buffered saline
TRPV4: Transient receptor potential cation channel subfamily V member 4

## References

[1] Lee WH, Choong LY, Jin TH, Mon NN, Chong S, Liew CS, Putti T, Lu SY, Harteneck C, Lim YP (2017). TRPV4 plays a role in breast cancer cell migration via Ca2+−dependent activation of AKT and downregulation of E-cadherin cell cortex protein. Oncogene.

[2] Clapham DE, Montell C, Schultz G, Julius D, International Union of P (2003). International Union of Pharmacology. XLIII. Compendium of voltage-gated ion channels: transient receptor potential channels. Pharmacol Rev.

[3] Antigny F, Girardin N, Frieden M (2012). Transient receptor potential canonical channels are required for in vitro endothelial tube formation. J Biol Chem.

[4] Kwan HY, Huang Y, Yao X (2007). TRP channels in endothelial function and dysfunction. Biochim Biophys Acta.

[5] Bihari S, Dixon DL, Lawrence MD, De Bellis D, Bonder CS, Dimasi DP, Bersten AD. Fluid-induced lung injury-role of TRPV4 channels. Pflugers Arch. 2017;469(9):1121–34. 10.1007/s00424-017-1983-1 28456852

[6] Naik JS, Osmond JM, Walker BR, Kanagy NL (2016). Hydrogen sulfide-induced vasodilation mediated by endothelial TRPV4 channels. Am J Physiol Heart Circ Physiol.

[7] Dragoni S, Guerra G, Pla AF, Bertoni G, Rappa A, Poletto V, Bottino C, Aronica A, Lodola F, Cinelli MP (2015). A functional transient receptor potential Vanilloid 4 (TRPV4) channel is expressed in human endothelial progenitor cells. J Cell Physiol.

[8] Suresh K, Servinsky L, Reyes J, Baksh S, Undem C, Caterina M, Pearse DB, Shimoda LA (2015). Hydrogen peroxide-induced calcium influx in lung microvascular endothelial cells involves TRPV4. Am J Physiol Lung Cell Mol Physiol.

[9] Monaghan K, McNaughten J, McGahon MK, Kelly C, Kyle D, Yong PH, McGeown JG, Curtis TM (2015). Hyperglycemia and diabetes Downregulate the functional expression of TRPV4 channels in retinal microvascular endothelium. PLoS One.

[10] Supplemental therapeutic oxygen for Prethreshold retinopathy of prematurity (STOP-ROP), a randomized, controlled trial. I: primary outcomes. Pediatrics. 2000;105(2):295–310. 10.1542/peds.105.2.295 10654946

[11] Abcouwer SF. Angiogenic factors and cytokines in diabetic retinopathy. J Clin Cell Immunol. 2013;Suppl 1(11):1–12. 10.4172/2155-9899 PMC3852182 24319628

[12] Berger AR, Cruess AF, Altomare F, Chaudhary V, Colleaux K, Greve M, Kherani A, Mandelcorn ED, Parsons H, Rheaume MA (2015). Optimal treatment of retinal vein occlusion: Canadian expert consensus. Ophthalmologica.

[13] Ma X, Qiu S, Luo J, Ma Y, Ngai CY, Shen B, Wong CO, Huang Y, Yao X (2010). Functional role of vanilloid transient receptor potential 4-canonical transient receptor potential 1 complex in flow-induced Ca2+ influx. Arterioscler Thromb Vasc Biol.

[14] Sonkusare SK, Bonev AD, Ledoux J, Liedtke W, Kotlikoff MI, Heppner TJ, Hill-Eubanks DC, Nelson MT (2012). Elementary Ca2+ signals through endothelial TRPV4 channels regulate vascular function. Science.

[15] Ma X, Du J, Zhang P, Deng J, Liu J, Lam FF, Li RA, Huang Y, Jin J, Yao X (2013). Functional role of TRPV4-KCa2.3 signaling in vascular endothelial cells in normal and streptozotocin-induced diabetic rats. Hypertension.

[16] Zhang FF, Zhu YF, Zhao QN, Yang DT, Dong YP, Jiang L, Xing WX, Li XY, Xing H, Shi M (2014). Microvesicles mediate transfer of P-glycoprotein to paclitaxel-sensitive A2780 human ovarian cancer cells, conferring paclitaxel-resistance. Eur J Pharmacol.

[17] Yap FC, Weber DS, Taylor MS, Townsley MI, Comer BS, Maylie J, Adelman JP, Lin MT (2016). Endothelial SK3 channel-associated Ca2+ microdomains modulate blood pressure. Am J Physiol Heart Circ Physiol.

[18] Jiang L, He D, Yang D, Chen Z, Pan Q, Mao A, Cai Y, Li X, Xing H, Shi M (2014). MiR-489 regulates chemoresistance in breast cancer via epithelial mesenchymal transition pathway. FEBS Lett.

[19] Faibish M, Francescone R, Bentley B, Yan W, Shao R (2011). A YKL-40-neutralizing antibody blocks tumor angiogenesis and progression: a potential therapeutic agent in cancers. Mol Cancer Ther.

[20] Li Y, Yang X, Wu Y, Zhao K, Ye Z, Zhu J, Xu X, Zhao X, Xing C. B7-H3 promotes gastric cancer cell migration and invasion. Oncotarget. 2017;8(42):71725–35. 10.18632/oncotarget.17847 PMC5641084 29069741

[21] Ma X, He D, Ru X, Chen Y, Cai Y, Bruce IC, Xia Q, Yao X, Jin J (2012). Apigenin, a plant-derived flavone, activates transient receptor potential vanilloid 4 cation channel. Br J Pharmacol.

[22] Pan Z, Yang H, Mergler S, Liu H, Tachado SD, Zhang F, Kao WW, Koziel H, Pleyer U, Reinach PS (2008). Dependence of regulatory volume decrease on transient receptor potential vanilloid 4 (TRPV4) expression in human corneal epithelial cells. Cell Calcium.

[23] Martin E, Dahan D, Cardouat G, Gillibert-Duplantier J, Marthan R, Savineau JP, Ducret T (2012). Involvement of TRPV1 and TRPV4 channels in migration of rat pulmonary arterial smooth muscle cells. Pflugers Arch.

[24] Mergler S, Valtink M, Takayoshi S, Okada Y, Miyajima M, Saika S, Reinach PS (2014). Temperature-sensitive transient receptor potential channels in corneal tissue layers and cells. Ophthalmic Res.

[25] Alkozi HA, Pintor J (2015). TRPV4 activation triggers the release of melatonin from human non-pigmented ciliary epithelial cells. Exp Eye Res.

[26] Martinez-Rendon J, Sanchez-Guzman E, Rueda A, Gonzalez J, Gulias-Canizo R, Aquino-Jarquin G, Castro-Munozledo F, Garcia-Villegas R (2017). TRPV4 regulates tight junctions and affects differentiation in a cell culture model of the corneal epithelium. J Cell Physiol.

[27] Aase K, Ernkvist M, Ebarasi L, Jakobsson L, Majumdar A, Yi C, Birot O, Ming Y, Kvanta A, Edholm D (2007). Angiomotin regulates endothelial cell migration during embryonic angiogenesis. Genes Dev.

[28] Ucuzian AA, Greisler HP (2007). In vitro models of angiogenesis. World J Surg.

[29] Taylor L, Arner K, Ghosh F (2017). Specific inhibition of TRPV4 enhances retinal ganglion cell survival in adult porcine retinal explants. Exp Eye Res.

[30] Ryskamp DA, Frye AM, Phuong TT, Yarishkin O, Jo AO, Xu Y, Lakk M, Iuso A, Redmon SN, Ambati B (2016). TRPV4 regulates calcium homeostasis, cytoskeletal remodeling, conventional outflow and intraocular pressure in the mammalian eye. Sci Rep.

