# Supplementary material for: TRPV4 regulates migration and tube formation of human retinal capillary endothelial cells
Source: BMC Ophthalmol. 2018 Feb 12;18:38. doi: 10.1186/s12886-018-0697-2 (PMC5809855; doi:10.1186/s12886-018-0697-2)
Supplement: Supplementary file 1 — Raw materials of western blot, what I used is framed in the red box. Raw materials of transwell and tube formation, every first picture is what I used in the article. (PPTX 10465 kb) [file 12886_2018_697_MOESM1_ESM.pptx]

## Slide 1
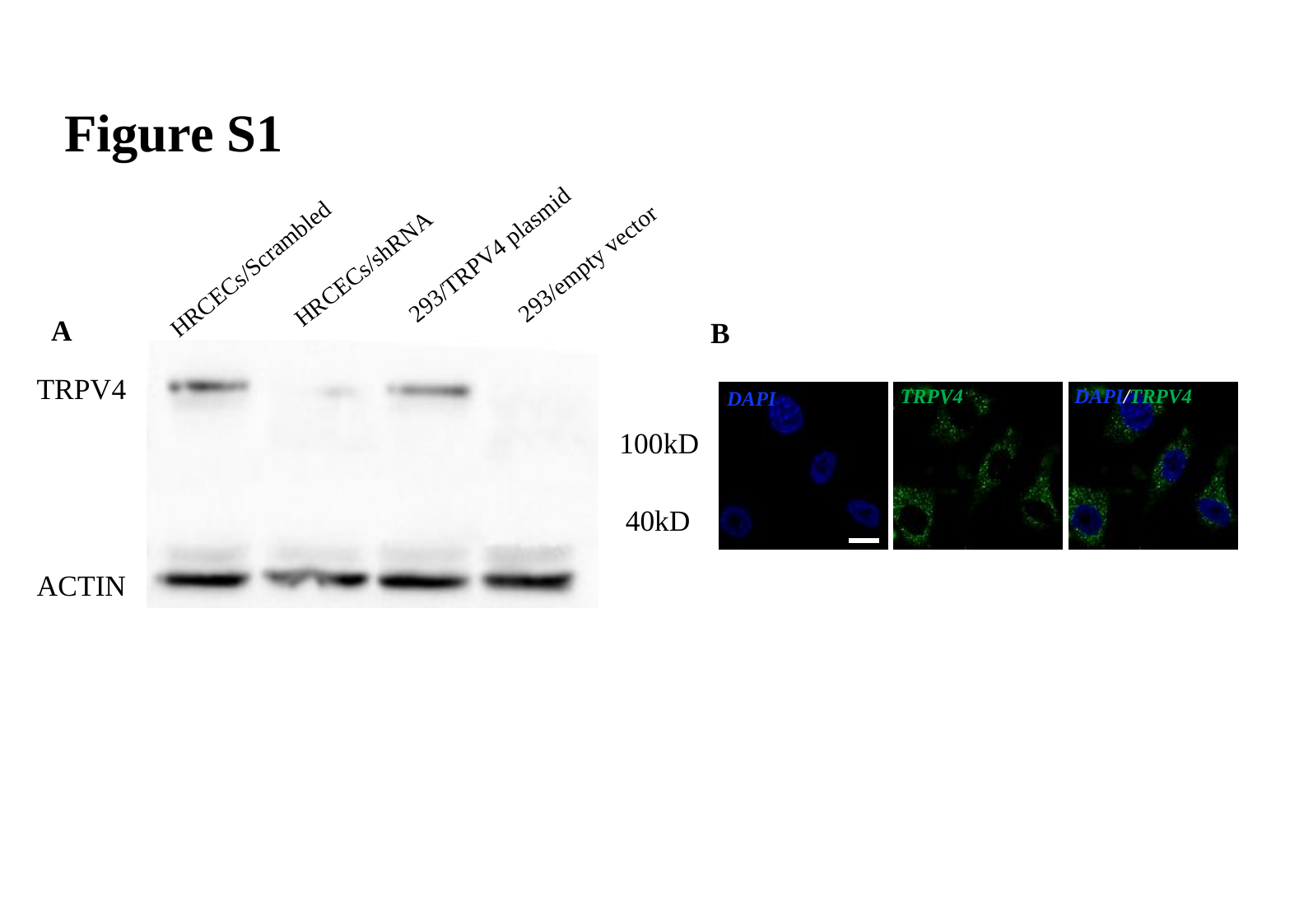

Figure S1
293/TRPV4 plasmid
293/empty vector
HRCECs/shRNA
HRCECs/Scrambled
A
B
TRPV4
TRPV4
DAPI/TRPV4
DAPI
100kD
40kD
ACTIN

## Slide 2
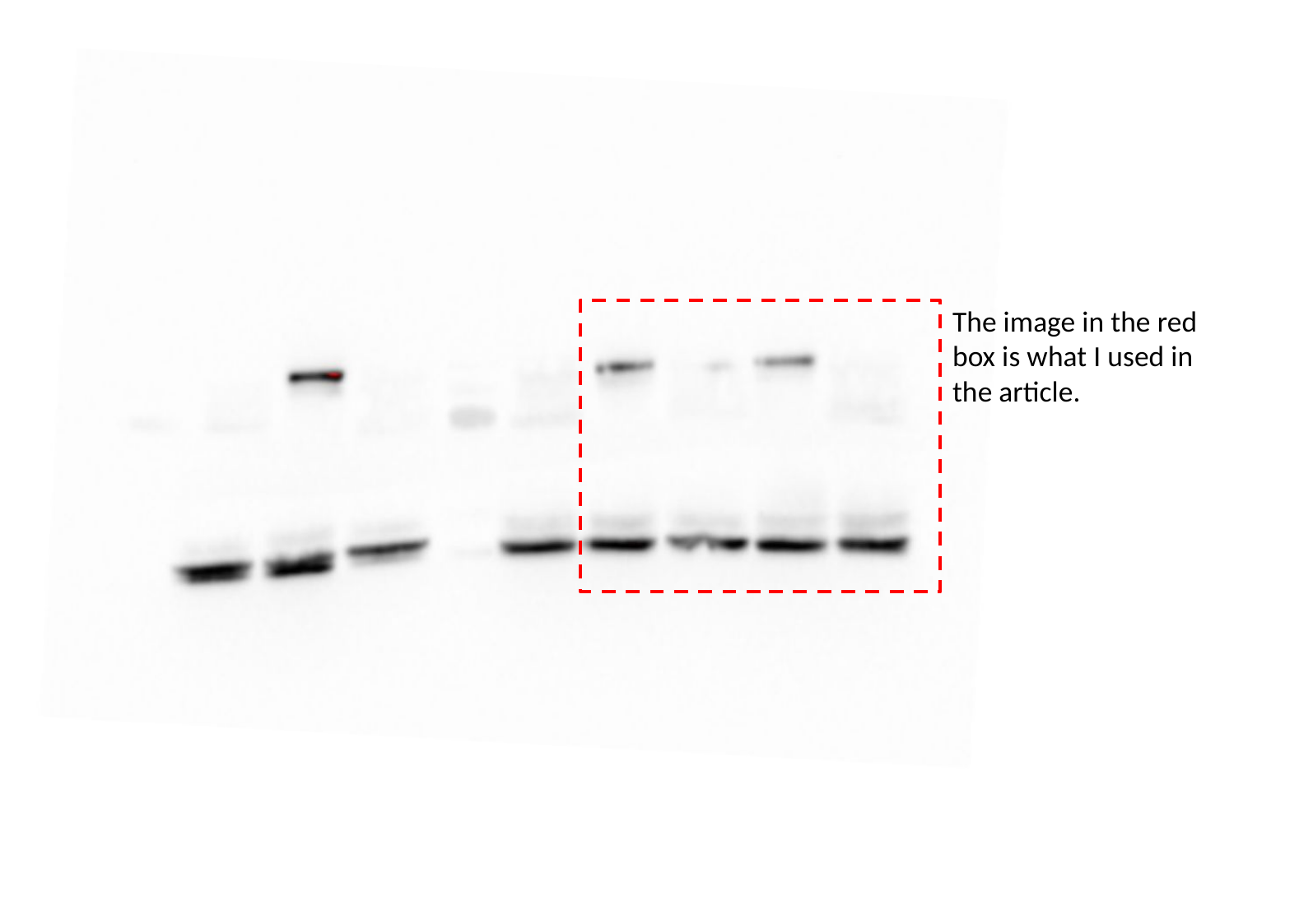

The image in the red box is what I used in the article.

## Slide 3
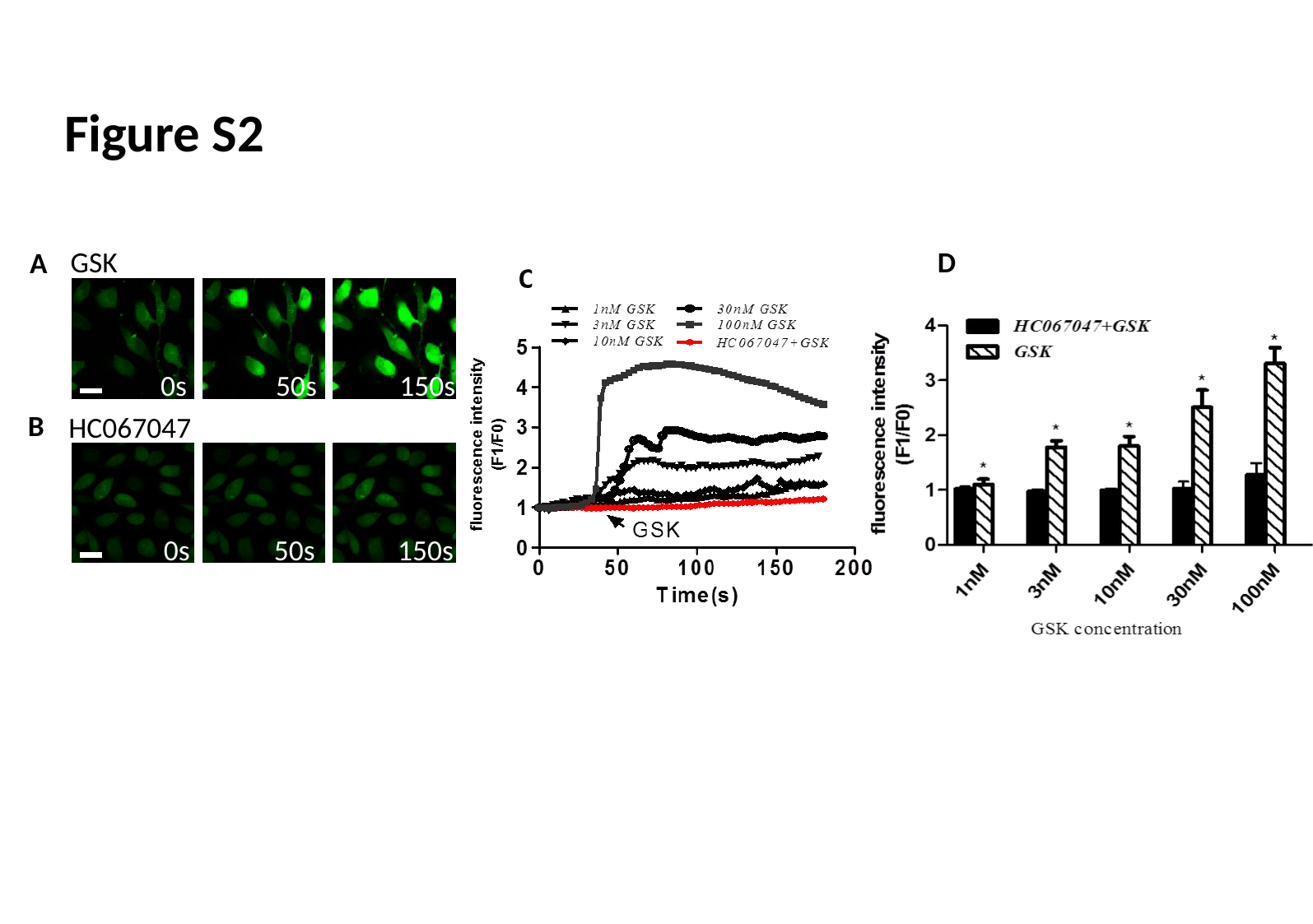

Figure S2
GSK
A
0s
50s
150s
HC067047
 0s
 50s
150s
D
B

## Slide 4
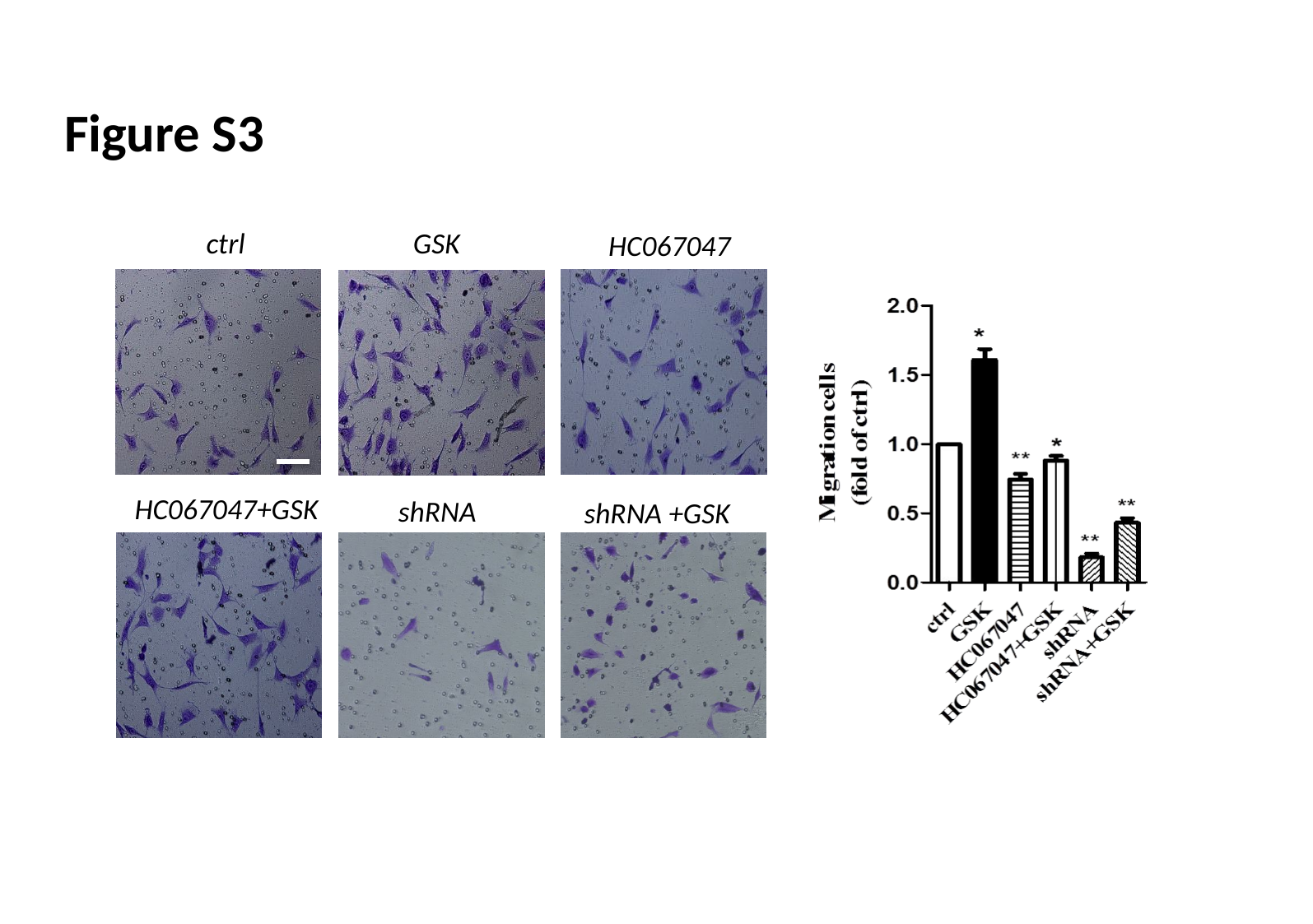

Figure S3
ctrl
GSK
HC067047
HC067047+GSK
shRNA
shRNA +GSK

## Slide 5
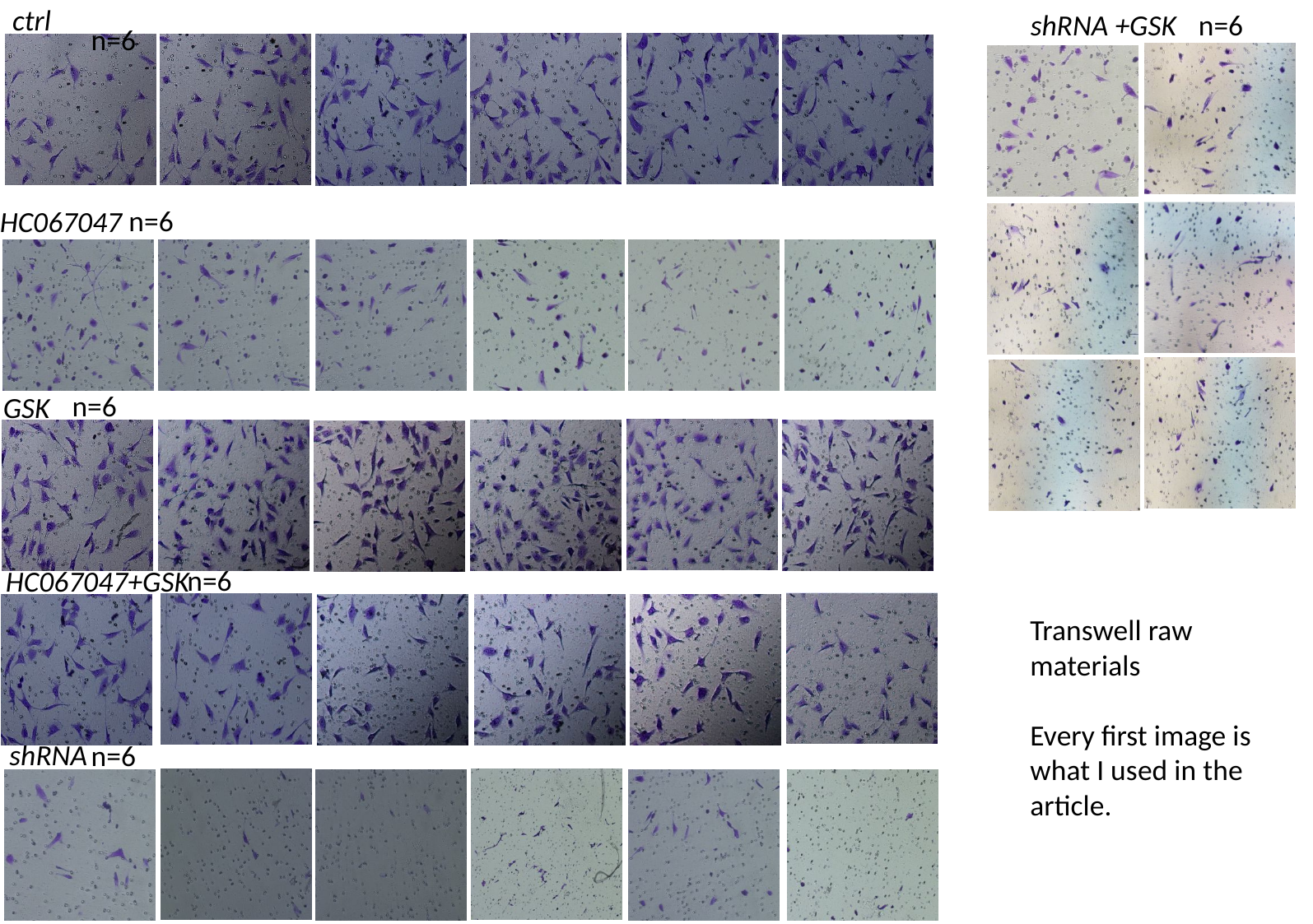

ctrl
shRNA +GSK
n=6
n=6
n=6
HC067047
n=6
GSK
n=6
HC067047+GSK
Transwell raw materials
Every first image is what I used in the article.
shRNA
n=6

## Slide 6
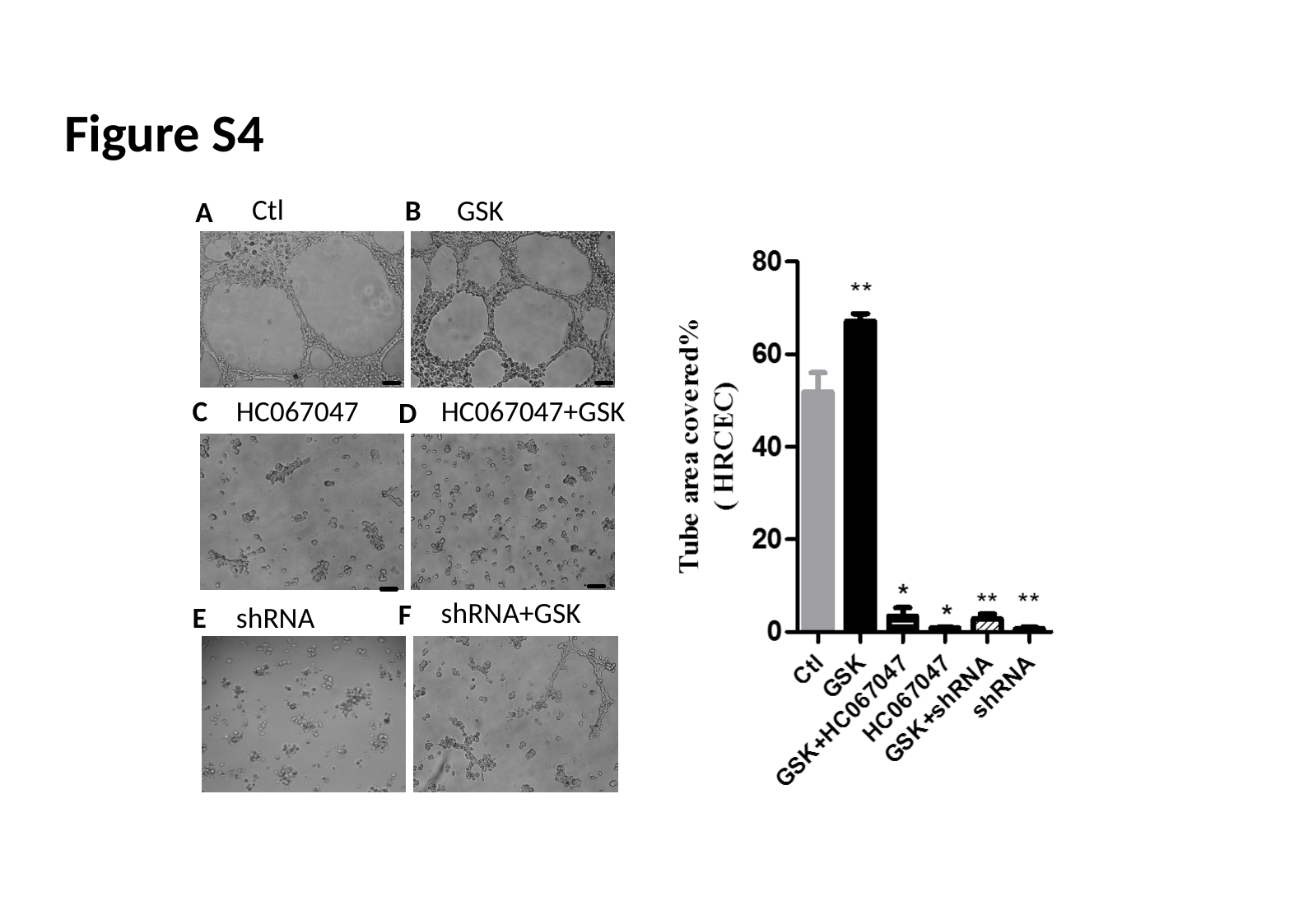

Figure S4
Ctl
B
GSK
A
C
HC067047
HC067047+GSK
D
shRNA+GSK
F
E
shRNA

## Slide 7
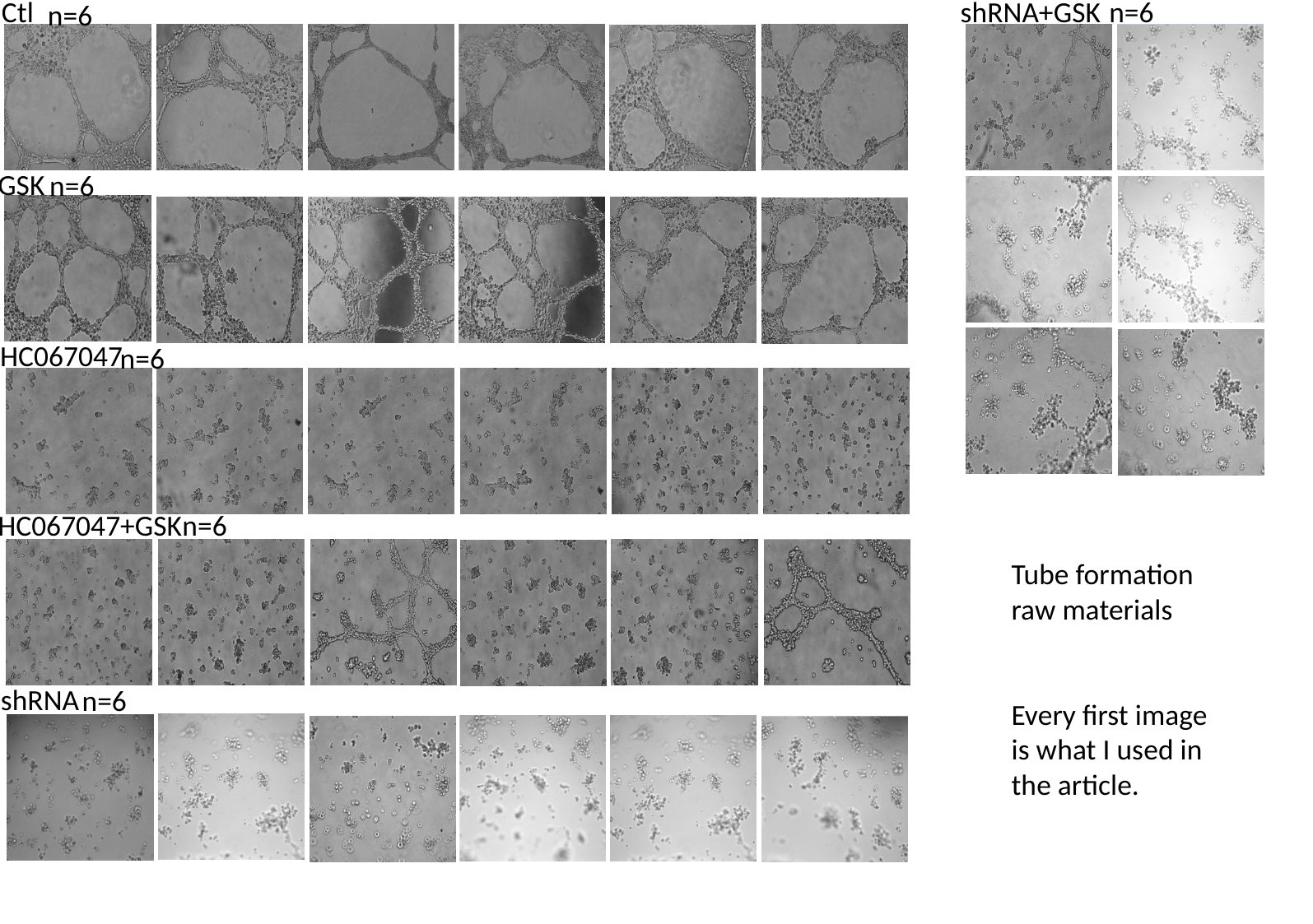

Ctl
n=6
shRNA+GSK
n=6
GSK
n=6
HC067047
n=6
HC067047+GSK
n=6
Tube formation raw materials
Every first image is what I used in the article.
shRNA
n=6
